# Combined conservative hemodynamic treatment for venous insufficiency strategy and negative-pressure wound therapy for the management of a refractory venous ulcer: a case report and review of the literature

**DOI:** 10.1186/s13256-026-06074-z

**Published:** 2026-05-21

**Authors:** Chunyan Li, Youli Wang, Shiyan Ren

**Affiliations:** 1 Operating Room, Aviation General Hospital, No. 3 Anwai Beiyuan, Chaoyang District, Beijing, 100012 China; 2 Department of General Surgery, Aviation General Hospital, No. 3 Anwai Beiyuan, Chaoyang District, Beijing, 100012 China

**Keywords:** Chronic venous ulcer, Varicose veins, CHIVA, Negative pressure wound therapy, Case report

## Abstract

**Background:**

Chronic venous ulcers (CVUs), affecting 1–5% of adults, present significant management challenges due to delayed healing and high recurrence rates. Negative pressure wound therapy (NPWT) has emerged as a beneficial adjunct to compression therapy for refractory cases.

**Case presentation:**

A 78-year-old Chinese female with a 30-year history of symptomatic bilateral varicose veins CEAP C6 (CEAP classification stands for Clinical (C), Etiological (E), Anatomical (A), and Pathophysiological (P)) presented with two recalcitrant venous ulcers (5 cm × 8 cm and 1 cm × 2 cm) on the right lateral malleolus. The ulcers were characterized by necrotic tissue, seropurulent exudate, and peri-wound hyperpigmentation. Previous treatment, including a skin graft, had failed. She was diagnosed with varicose vein C6, chronic venous ulcers, skin infection and anemia. A multimodal management strategy was implemented, comprising surgical correction of venous insufficiency using the CHIVA strategy, radical debridement, systemic antibiotics, NPWT, and sustained compression therapy. Serial documentation showed progressive wound closure. Her ulcers were completely healed on day 75 after initial treatment, with no recurrence on 11-month follow-up. No recurrence was observed at the 11-month follow-up.

**Conclusion:**

This case demonstrates that combining the CHIVA strategy with NPWT within a multidisciplinary care framework can effectively overcome healing barriers in complex, refractory CVUs. This approach addresses the underlying etiology while optimizing the local wound environment, leading to accelerated healing and prevention of recurrence.

## Introduction

Chronic venous ulcers (CVUs), affecting approximately 1–5% of the global adult population, result from sustained venous hypertension and chronic inflammation [1]. Compression therapy remains the cornerstone first-line treatment; however, about 30–50% of CVUs remain unhealed at 24 weeks, underscoring the need for advanced adjuvant therapies [2]. Negative pressure wound therapy (NPWT) enhances healing by mitigating edema, stimulating granulation tissue formation, and removing infectious exudate [3].

One surgical strategy for managing varicose veins secondary to chronic venous insufficiency is the Conservative Hemodynamic treatment for Venous Insufficiency (CHIVA) strategy. This approach, based on precise venous hemodynamic mapping, aims to correct pathological reflux while preserving the superficial venous system, thereby addressing the root cause of venous hypertension [4].

This report presents the case of a 78-year-old female with a 30-year history of varicose veins and two refractory CVUs that had failed previous treatments, including a skin graft. We successfully managed this complex case using a multimodal protocol integrating the CHIVA strategy, NPWT, radical debridement, targeted antibiotics, and nutritional support. We highlight the synergistic effects of this comprehensive approach.

## Case presentation

A 78-year-old Chinese female presented to our vascular clinic on October 8, 2024, with recurrent skin infections and two non-healing ulcers on the right lateral malleolus. She had a 30-year history of symptomatic bilateral varicose veins, classified as CEAP C6. Comorbidities included iron-deficiency anemia (hemoglobin: 98 g/L). She had no history of diabetes, hypertension, smoking, or alcohol use. Her family history was unremarkable.

For two years, she had experienced constant discomfort and intermittent pain from the ulcers. She had used herbal paste without success and had undergone a skin graft two years prior, which failed one year later. The pain limited her ambulation, leading to significant disappointment and depressive mood. She was persuaded by her daughter to seek further treatment.

**Fig. 1:**
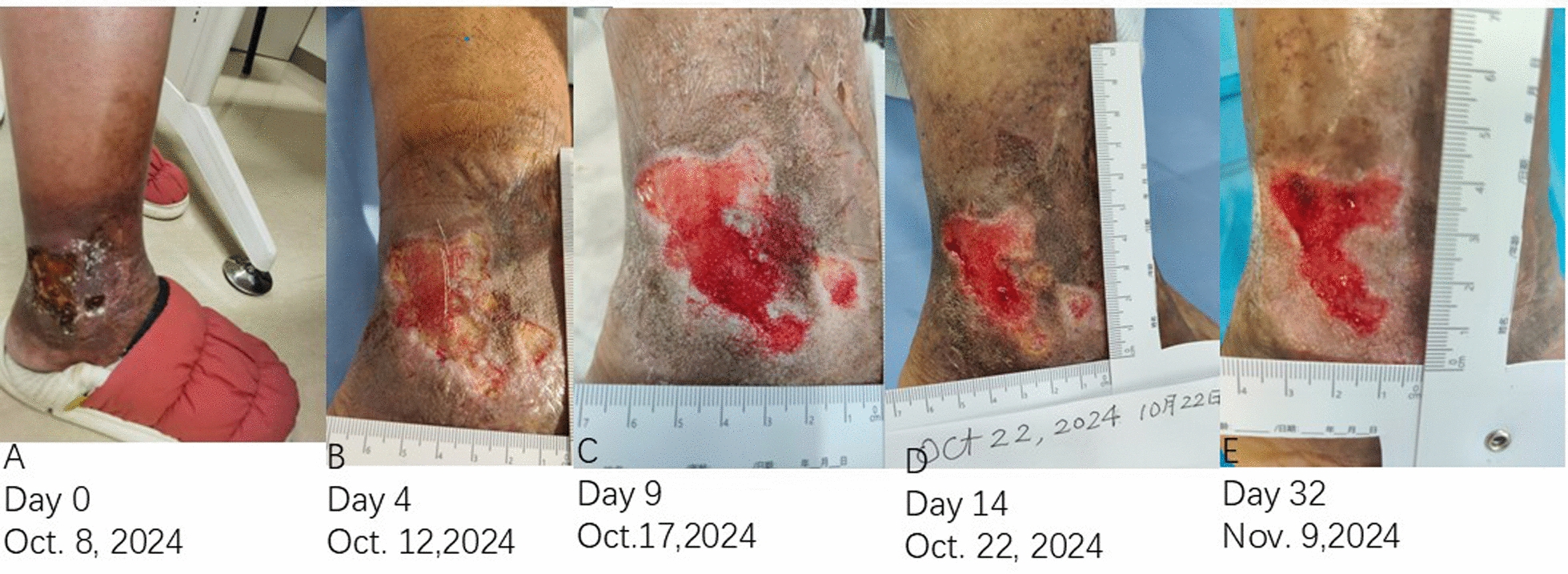
Clinical progression during the initial treatment phase. **A** Day 0: presentation showing the primary ulcer with necrotic base and exudate. **B** Day 4: early robust granulation tissue formation after debridement and negative pressure wound therapy initiation. **C** Day 9: continued progression of granulation tissue. **D** Day 14: advanced healing with epithelialization. **E** Day 32: progressive epithelial closure

On admission, her vital signs were normal. Physical examination revealed two foul-smelling cutaneous ulcers on the right lower extremity. The primary ulcer was large (5.0 cm × 8.0 cm) and irregularly shaped with a depth of 4 mm, featuring a necrotic, fibrinous base covered with seropurulent exudate. A smaller secondary ulcer (1.0 cm × 2.0 cm) was located adjacent to the lateral malleolus. The peri-wound skin exhibited significant hyperpigmentation, hemosiderin deposition, and induration, consistent with long-standing venous insufficiency (Fig. 1). Her pedal pulses were palpable.

Laboratory findings upon hospitalization were as follows: RBC 3.41 × 10^12^/L, WBC 4.72 × 10⁹/L, hemoglobin 105 g/L, platelets 236 × 10⁹/L, ferritin 177.9 ng/mL, folic acid 4.25 ng/mL, vitamin B12 511 pg/mL, transferrin 1.67 g/L, total iron-binding capacity 40 µmol/L, iron 10.7 µmol/L, neutrophils 4.00 × 10⁹/L. Serum total protein was 59.2 g/L (low), and albumin was 36.1 g/L (low). Duplex ultrasound confirmed saphenofemoral junction (SFJ) reflux and varicose veins.

*Final diagnoses* Chronic venous dysfunction (CEAP C6), venous ulcers, skin infection, and iron-deficiency anemia.

## Management

A comprehensive, multimodal management strategy was implemented.

### Correction of venous hemodynamic insufficiency with CHIVA

Under local anesthesia, the patient underwent the CHIVA strategy. Guided by ultrasonographic mapping, the SFJ reflux points were selectively disconnected to normalize venous hemodynamics and reduce ambulatory venous pressure [4], thereby targeting the fundamental cause of ulceration.

### Wound debridement and infection control

Aggressive weekly sharp and mechanical debridement was performed to remove necrotic tissue and exudate until a viable wound bed was established. Intravenous piperacillin–tazobactam (4.5 g every 8 hours) was administered for 7 days. Following clinical improvement, therapy was de-escalated to oral ciprofloxacin (500 mg every 12 hours) based on culture sensitivities. Warfarin (3 mg daily) was administered for thrombosis prophylaxis, with a target INR of 2.0–3.0.

### Advanced wound therapy and compression

NPWT was applied using a polyurethane foam dressing with continuous negative pressure set at − 120 mmHg [5]. Dressings were changed every 5–7 days. Sustained compression therapy was maintained throughout the treatment period.

### Adjunctive systemic and supportive therapies

Oral iron supplementation (ferrous sulfate 325 mg daily) was prescribed to correct anemia. A supervised ambulation protocol was implemented to enhance calf muscle pump function and venous return.

## Outcomes and follow-up

**Fig. 2:**
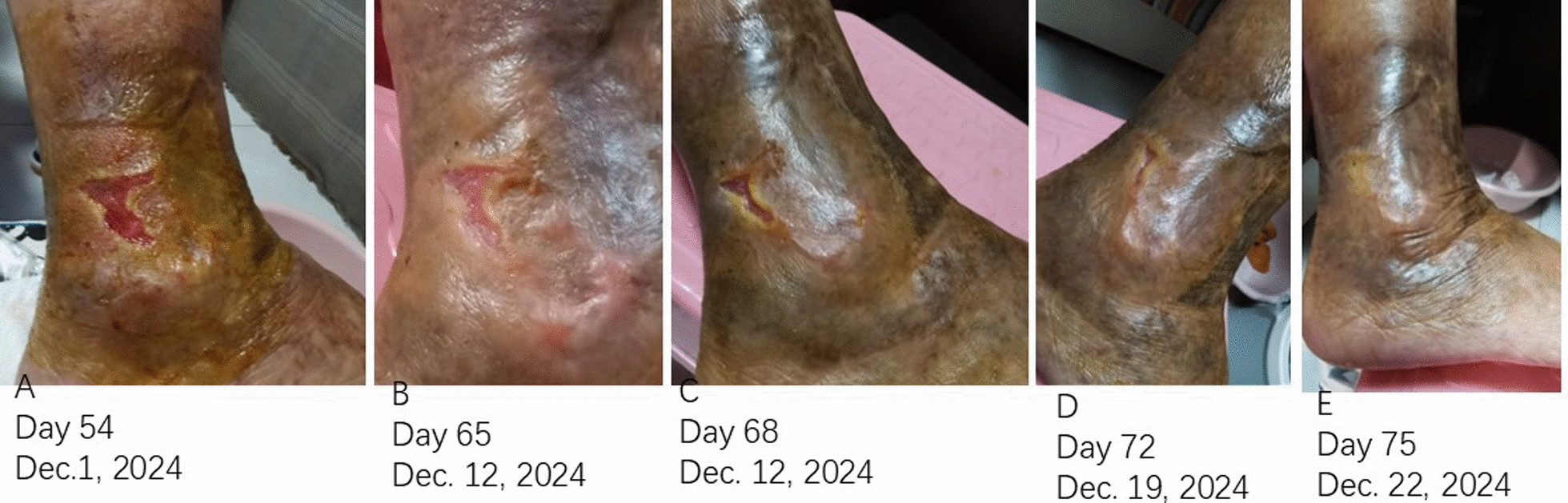
Scar maturation and wound closure. **A** Day 54: healed site with erythematous scar. **B** Day 65: reduced erythema and normalized texture. **C** Day 68: further resolution of redness. **D** Day 72: advanced scar maturation. **E** Day 75: final outcome showing a mature, supple scar

The patient exhibited a markedly positive and rapid response. Serial photographic documentation illustrated the healing trajectory (Figs. 1 and 2). Robust granulation tissue was evident by Day 4 (Fig. 1B). Epithelialization exceeding 70% was observed by Day 14 (Fig. 1D). A mature, pliable scar with normalized pigmentation and resolved induration was noted on Day 75 (Fig. 2E). At the 11-month follow-up, there was no recurrence. The patient resumed normal ambulation without discomfort, and her mood significantly improved.

## Discussion

This case demonstrates the successful management of a refractory CVU through an integrative strategy that concurrently addresses the local wound environment and the underlying venous pathology. The rapid healing underscores the efficacy of combining NPWT with definitive surgical correction.

A pivotal aspect was the early use of the CHIVA strategy to correct SFJ reflux, the root cause of venous hypertension. This aligns with modern principles advocating for the treatment of superficial venous incompetence in patients with active ulceration [6]. By normalizing venous pressure, CHIVA creates a favorable environment for healing [4].

NPWT significantly enhanced the local wound conditions. Evidence suggests NPWT can increase the healing rate of CVUs by approximately 34% compared to standard moist wound therapy [7]. Its mechanisms—macrostrain promoting angiogenesis and granulation [8], bacterial clearance reducing bioburden [9, 10], and edema reduction improving microcirculation [7]—worked synergistically with targeted antibiotics and surgical correction.

This case reinforces that controlling infection, correcting underlying reflux, and addressing systemic factors like anemia are critical. The traditional “wound-care only” approach is often insufficient. Lifelong compression therapy remains essential for preventing recurrence [11]. Ultimately, managing complex CVUs requires a dedicated, multidisciplinary team.

### Limitations

The primary limitations of this case report are inherent to its design as a single-case study, which limits the generalizability of the findings. The lack of a comparative control group makes it difficult to isolate the individual contributions of the CHIVA and NPWT interventions. Furthermore, the 11-month follow-up, while promising, is relatively short for assessing the long-term recurrence risk of venous ulcers, and the positive outcome may be influenced by patient-specific factors such as compliance.

## Conclusion

NPWT significantly accelerates the healing of refractory CVUs when integrated into a multimodal management strategy that includes surgical correction of venous insufficiency (CHIVA), rigorous debridement, targeted infection control, and sustained compression. This case provides clear visual documentation of the healing trajectory and serves as a practical reference for clinicians, demonstrating that a comprehensive, etiology-driven approach is essential for achieving optimal outcomes.

## Data Availability

The data and related information are available from the corresponding author upon reasonable request.

## Abbreviations

CHIVA: Conservative Hemodynamic treatment for Venous Insufficiency
NPWT: Negative pressure wound therapy
CVUs: Chronic venous ulcers
SFJ: Saphenofemoral junction
CEAP: Clinical (C), Etiological (E), Anatomical (A), and Pathophysiological (P)

## References

[1] Robertson L, Evans C, Fowkes FG. Epidemiology of chronic venous disease. Phlebology. 2008;23(3):103–11. 18467617 10.1258/phleb.2007.007061

[2] O’Meara S, Cullum N, Nelson EA, Dumville JC. Compression for venous leg ulcers. Cochrane Database Syst Rev. 2012. 10.1002/14651858.CD000265.pub3. 23152202 10.1002/14651858.CD000265.pub3 PMC7068175

[3] Schwien T, Gilbert J, Lang C. Pressure ulcer prevalence and the role of negative pressure wound therapy in reducing incidence and patient outcomes. Ostomy Wound Manage. 2005;51(9):62–8. 16230764

[4] Franceschi C, Bahnini A. CHIVA strategy: a paradigm shift in the treatment of varicose veins. J Vasc Surg Venous Lymphat Disord. 2020;8(4):676–85.32444277

[5] Partsch H. Compression therapy: clinical and experimental evidence. Ann Vasc Dis. 2012;5(4):416–22. 23641263 10.3400/avd.ra.12.00068 PMC3641539

[6] Gohel MS, Heatley F, Liu X, *et al*. A randomized trial of early endovenous ablation in venous ulceration. N Engl J Med. 2018;378(22):2105–14. 29688123 10.1056/NEJMoa1801214

[7] Xie X, McGregor M, Dendukuri N. The clinical effectiveness of negative pressure wound therapy: a systematic review. J Wound Care. 2020;29(Sup3a):S1–26. 10.12968/jowc.2010.19.11.79697 21135797

[8] Saxena V, Hwang CW, Huang S, Eichbaum Q, Ingber D, Orgill DP. Vacuum-assisted closure: microdeformations of wounds and cell proliferation. Plast Reconstr Surg. 2004;114(5):1086–96. 15457017 10.1097/01.prs.0000135330.51408.97

[9] Mouës CM, van den Bemd GJ, Heule F, Hovius SE. Comparing conventional gauze therapy to vacuum-assisted closure wound therapy: a prospective randomised trial. J Plast Reconstr Aesthet Surg. 2007;60(6):672–81. 17485058 10.1016/j.bjps.2006.01.041

[10] Braakenburg A, Obdeijn MC, Feitz R, van Rooij IA, van Griethuysen AJ, Klinkenbijl JH. The clinical efficacy and cost-effectiveness of the vacuum-assisted closure technique in the management of acute and chronic wounds: a randomized controlled trial. Plast Reconstr Surg. 2006;118(2):390–7. 16874208 10.1097/01.prs.0000227675.63744.af

[11] Nelson EA, Bell-Syer SEM. Compression for preventing recurrence of venous ulcers. Cochrane Database Syst Rev. 2014. 10.1002/14651858.CD002303.pub3. 25203307 10.1002/14651858.CD002303.pub3 PMC7138196

